# The Effects of Cognitive Training on Executive Function and Cognition

**DOI:** 10.3390/brainsci15030272

**Published:** 2025-03-04

**Authors:** Špela Bogataj, Bart Roelands

**Affiliations:** 1 Department of Nephrology, University Medical Center Ljubljana, 1000 Ljubljana, Slovenia; 2 Faculty of Sport, University of Ljubljana, 1000 Ljubljana, Slovenia; 3 Human Physiology and Sports Physiotherapy Research Group, Faculty of Physical Education and Physiotherapy, Vrije Universiteit Brussel, 1050 Brussels, Belgium; bart.roelands@vub.be; 4 BruBotics, Vrije Universiteit Brussel, 1050 Brussels, Belgium

Cognitive training has emerged as a promising approach to enhancing executive function and cognition across various populations from children to older adults [1,2,3]. Given the increasing prevalence of cognitive decline and neurodegenerative conditions, understanding the mechanisms and effectiveness of cognitive training interventions is crucial [4,5]. Over the past decades, research has demonstrated the benefits of targeted cognitive exercises, but many questions remain regarding optimal training protocols, the durability of effects, and the interaction between cognitive training and other interventions such as physical exercise [5,6].

Despite progress in this field, significant knowledge gaps persist. These include the need for a deeper understanding of individualized training responses, the role of neuroplasticity in long-term cognitive improvements, and the applicability of cognitive training in real-world settings [7,8]. Furthermore, the interaction between cognitive training and lifestyle factors, including physical activity, social engagement, and environmental influences, remains an area requiring further investigation [9,10]. Additionally, while technology-driven cognitive training programs, including virtual reality and AI-assisted tools, have gained attention, their efficacy compared to traditional cognitive training approaches is still under debate [11,12].

This Special Issue of Brain Sciences addresses several of these gaps by presenting a diverse range of studies exploring the impact of cognitive training on executive function and cognition. The papers included examine innovative intervention strategies across different populations and contexts. Contributions include the evaluation of AI chatbot interactions for enhancing executive functions, the benefits of board games in improving visuospatial memory and mathematical skills in children, and the impact of aquatic exercise on cognitive function in older adults. Additionally, this Issue explores the relationship between prior physical activity engagement and quality of life perceptions, the effectiveness of parental guidance in self-regulation interventions for children, and the cognitive effects of virtual reality exergames compared to nature exposure.

By integrating systematic reviews, controlled clinical trials, and experimental methodologies, this Special Issue provides comprehensive insights into how cognitive training interventions can be optimized for short- and long-term benefits. The findings suggest promising avenues for future research, such as the development of AI-assisted cognitive training tools, the integration of physical and cognitive training for older adults, and the role of structured play in cognitive development among children.

Looking ahead, research should focus on personalized cognitive training interventions, leveraging technology to tailor programs based on individual cognitive profiles and needs. Large-scale longitudinal studies are also needed to assess the sustainability of training effects over time. Furthermore, interdisciplinary approaches combining neuroscience, psychology, and AI could lead to groundbreaking advancements in cognitive training methodologies. Additional exploration into the synergistic effects of cognitive training combined with social and/or physical activities may offer a more holistic approach to improve health and even performance. One example of such an innovative training methodology is Brain Endurance Training (BET), which integrates both physical and cognitive components within one training session. The rationale behind BET is that it leverages the combined effects of physical and cognitive training on brain function and structure, potentially producing synergistic benefits that enhance both general brain health and cognitive and physical performance capacity in athletes, in addition to patient populations [13]. This is nicely illustrated in a study by Bogataj et al. [14], in which a cohort of patients undergoing hemodialysis underwent a novel, 12-week, non-pharmacological intradialytic intervention combining physical and cognitive training, while a second group received standard care. This intervention led to improved physical and cognitive performance, preserved levels of brain-derived neurotrophic factor (BDNF), and better scores regarding mental fatigue, frailty, and quality of life [14].

We extend our gratitude to all the contributors who have enriched this Special Issue with their pioneering research. Their efforts enhance our understanding of cognitive training and its potential applications across different populations. We also acknowledge the reviewers whose expertise ensured the high quality of the published work.

As cognitive training research continues to evolve, we anticipate that future studies will refine and expand our knowledge, ultimately leading to more effective interventions for preserving and enhancing cognitive function across the lifespan. We hope this Special Issue serves as a foundation for ongoing exploration and innovation in this field.
